# Appropriate Medical Staffing Improves Patient Safety, Training and Doctor Wellbeing

**DOI:** 10.7759/cureus.32071

**Published:** 2022-11-30

**Authors:** Catriona Hunter, Amanda Thorne, Janet Fallon, Andrew J Stevenson

**Affiliations:** 1 Trauma and Orthopaedics, Musgrove Park Hospital, Taunton, GBR; 2 Breast Surgery, Musgrove Park Hospital, Taunton, GBR; 3 Respiratory Medicine, Musgrove Park Hospital, Taunton, GBR

**Keywords:** doctor wellbeing, workforce planning, out-of-hours patient care, postgraduate training, emergency cross-cover

## Abstract

Background

Cross-covering of medical and surgical specialities out-of-hours is a problem in many hospitals, leaving trainee doctors responsible out-of-hours for patients they have never met, in specialities where they do not normally work. This has implications for patient safety and doctor wellbeing. In our Trust, a historical decision resulted in trainee doctors in Trauma & Orthopaedics and Ear Nose and Throat Surgery being reallocated out-of-hours to cross-cover medical inpatients. This left one doctor cross-covering all surgical specialities, including General Surgery, Urology, Vascular, Ear, Nose and Throat surgery (ENT), Trauma & Orthopaedics (T&O) and Spinal Surgery. As the out-of-hours workload increased over time, this impacted negatively on patient safety and doctor wellbeing to a point where it became unsustainable.

Methods

Evidence of safety concerns relating to surgical night shifts was gathered from Exception Reporting data and anecdotally from the Postgraduate Doctor Forum. Once the scale of this problem was accepted by the hospital board, following the successful presentation of two Business Cases, 17 additional doctors were recruited. This recruitment reduced the cross-covering of specialities out-of-hours and enable adequate staffing throughout all departments. Qualitative evidence was gathered by surveying affected doctors before and after the change in order to assess its impact on doctor wellbeing, training and perceived patient safety. Quantitative analysis of Exception Reports and Immediate Safety Concerns was also performed.

Results

The survey results following the change were overwhelmingly positive, demonstrating a significant improvement in workload, rest breaks and quality of care for patients. Foundation doctors reported higher levels of confidence and enhanced training due to more consistent supervision. Job satisfaction improved, with 81% of surgical senior house officers reporting they would recommend their job, compared with 42% previously. Trends in out-of-hours Exception Reporting and patient safety concerns were analysed to show a moderate improvement following the intervention.

Conclusion

With the ever-increasing volume and complexity of patients presenting to global healthcare systems, it is key that staffing levels are safe and adequate in order to maintain patient safety and doctor wellbeing. This project has demonstrated how historic short-term fixes such as redeploying trainee doctors out of their home speciality and implementing cross-cover of multiple specialities can have detrimental long-term effects. Our preliminary data revealed multiple issues related to patient safety, junior doctor workload and lack of training opportunities. By using this data, and enlisting the help of multiple valued senior stakeholders, an acceptable Business Plan was approved by the Trust with a view to reversing these issues. The recruitment of additional Trust Grade doctors to create a third tier of the surgical out-of-hours cover has been instrumental in improving conditions within our Trust and has shown that adequate workforce planning is achievable when supported by robust evidence. This project could be used as a guide for other units seeking to make similar improvements.

## Introduction

Acute medical admissions in our Trust have climbed from 16,000 to 26,000 (62%) over the last 10 years. Somerset has an older and more rapidly ageing population when compared with average figures across the UK and this contributes to increasing medical complexity among our patient population, with the majority being frail with multiple co-morbidities [1]. As a result of this increasing medical workload, in 2006 a decision was made to reallocate Foundation Year 2 (FY2) doctors from Trauma & Orthopaedics (T&O) and Ear, Nose and Throat surgery (ENT) to provide ward cover for medical inpatients out-of-hours (OOH). This left one Core Surgical Trainee (CST) or equivalent providing emergency cross-cover for all surgical specialities, including General Surgery, Urology, Vascular Surgery, ENT, T&O and Spinal Surgery. The CST was supported OOH by a Foundation Year 1 (FY1) doctor and by speciality registrars or consultants off-site for each speciality.

Over time, patient volume continued to increase throughout the Trust, and feedback from surgical doctors suggested that this workload was becoming unsustainable and unsafe across the surgical directorate. The reallocation of surgical FY2 doctors to cross-cover medical patients OOH was also reported to negatively impact training for these doctors as a result of decreased time working in their own speciality. Feedback was collected from the Postgraduate Doctor Forum and Exception Reports, both developed through the 2016 Junior Doctor Contact [2]. Exception reporting is the means by which trainee doctors nationally can raise issues related to working hours, missed breaks and missed educational opportunities, and serves as a mechanism by which Trusts can monitor and intervene where working conditions do not meet acceptable standards [3]. Immediate Safety Concerns (ISCs) are a subset of ER that are thought to represent “immediate and substantive risk,” either to patients or to the doctor making the report [4].

The issues were identified by the Guardian of Safe Working (GOSW) and the Surgical Tutors in General Surgery and T&O, and the decision was made to pursue a resolution. This project was presented as a poster at the Bristol Patient Safety Conference, on 18th May 2022.

## Materials and methods

Preliminary data was gathered by the interrogation of ERs and ISCs raised within surgical specialities in the Trust between March 2019 and July 2021, and compared with those raised between August 2021 and July 2022.

Anonymised surveys were distributed to doctors affected by the situation in order to quantify the problem. These were circulated to all doctors providing OOH cross-cover of surgical specialities between 2019 and 2021. A similar survey was carried out of FY2s working in T&O during the same timeframe to analyse their perception of the working rota and its impact. Surveys were distributed via e-mail and social media. These also included free-text boxes to enable respondents to submit individual comments anonymously.

The GOSW and the surgical tutors used the evidence gathered to inform two Business Cases presented to the Trust Board [2]. The Business Cases proposed the recruitment of more doctors with a view to creating a second tier in the OOH surgical rota, in order to provide safe staffing levels OOH. The Business Cases were presented to the Trust Board in July 2020 and February 2021, both of which were successful.

By August 2021, recruitment was complete and the new rota was implemented. In total, 17 Trust Grade doctors were recruited. The successful recruitment of qualified, well-rounded doctors depended on the desirable nature of the jobs offered; typically a 60% clinical and 40% non-clinical split with a mandatory OOH commitment.

In the months following the change, further anonymised surveys were distributed to doctors working OOH in surgery and T&O in order to analyse the impact of the new working patterns. This included a number of trainee doctors who had worked in the directorate both before and after the change, allowing a direct comparison. ERs and ISCs continued to be monitored. The outcomes of these analyses were presented locally at the T&O Governance Meeting and regionally by the Director of Medical Education to the Deanery as an example of positive change.

## Results

Surveys

Anonymised surveys were conducted of trainee doctors working in surgery between 2019 and 2020. There were 12 responses from doctors who had cross-covered surgical specialities OOH, and 15 responses from T&O FY2 doctors who had cross-covered medical wards OOH. The results demonstrated that doctors covering surgical specialities OOH felt unsafe, unable to take breaks and generally would not recommend their job. The surveys showed that T&O FY2 doctors did not feel they had gained sufficient experience in T&O during their time working within the speciality.

**Figure 1:**
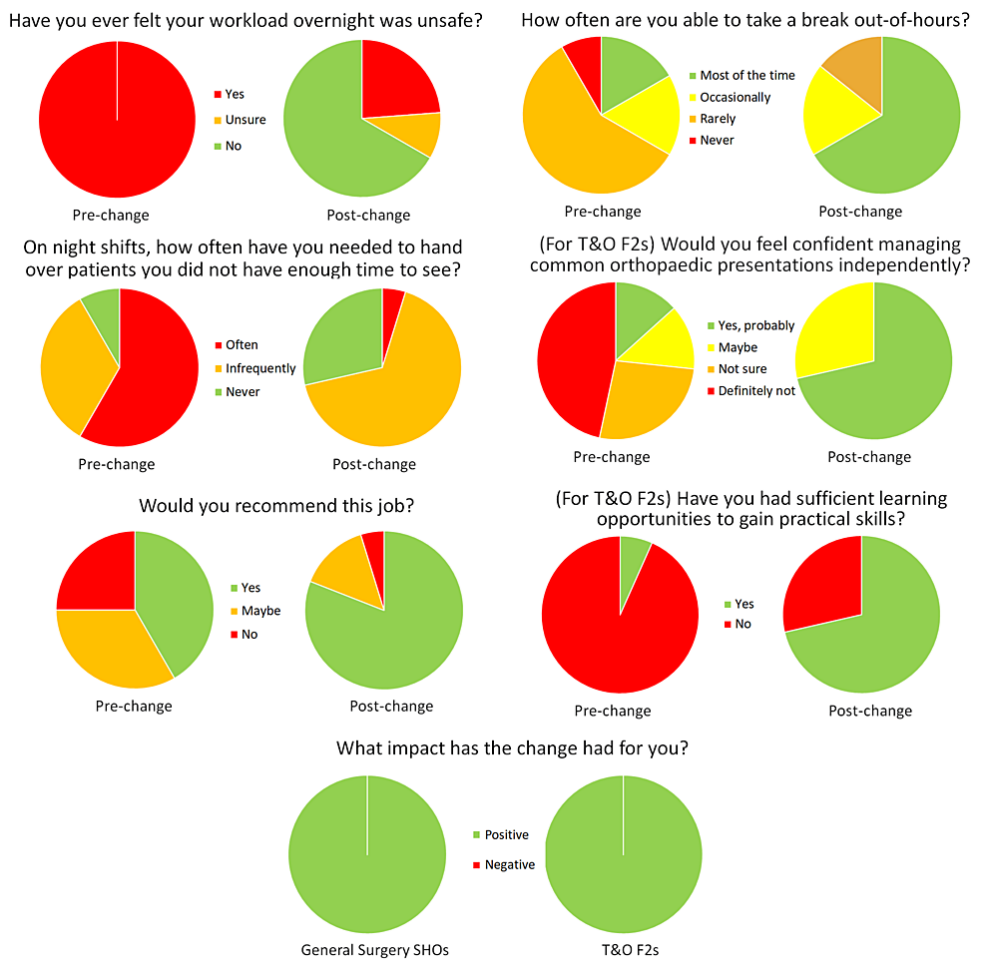
Pie-charts displaying responses to surveys before and after the intervention T&O: trauma and orthopaedics; OOH: out-of-hours; SHO: senior house officer (including Core Surgical Trainees and equivalent Trust Grade doctors); F2: Foundation Year 2 doctor

Following the changes, a second survey was distributed to doctors covering surgical specialities including T&O OOH between August 2021 and December 2021. There were 21 responses to this survey. The results of this survey were compared directly to the preliminary survey, showing significant improvements in conditions for doctors working OOH. The results of each set of surveys are summarised in Figure 1.

Some of the free-text responses are displayed in Table 1.

**Table 1:** Free-text responses from the surveys carried out before and after the intervention T&O: trauma and orthopaedics; OOH: out-of-hours

Pre-change	Post-change
“Unsafe, not well supported, shifts can be really busy and in situations you would be imminently needed in two different places”	“Covering T&O OOH allowed me to learn much more about T&O”
“I often felt one step away from making a possible error due to workload”	“I felt less overwhelmed”
“Busy but does provide good opportunity to learn situation awareness and time management”	“Night shifts are variable. Sometimes steady and manageable other time hectic and unsafe”
“I would recommend the job if T&O was covered by a separate doctor”	“Splitting up the workload means that patient care is better and safer”
“Cross cover of multiple specialities is not conducive to good learning”	“Enjoyed the job, good team, manageable night shifts”
“In view of the workload, because of receiving referrals from T&O in addition to the surgical patients, doing the nights is a lot of work”	“I would recommend the job but that does not mean the night shifts are easy—just that they are now better managed”
“Good training opportunity but extremely high patient load which is unsafe at times”	“Better continuity of care”

Exception reports

Between 2016 and 2020, over 80% of exception reports in the Trust came from Internal Medicine, General Surgery and T&O. These increased in number each year. The vast majority of these were related to hours worked, including lack of breaks and overtime. Some examples of ERs raised in the year prior to this project are shown in Table 2.

**Table 2:** Examples of exception reports highlighted prior to July 2021 BMA: British Medical Association; CST: core surgical trainee; T&O: trauma and orthopaedics

Exception reports
“No breaks taken in 13-hour shift due to workload. This is a recurrent problem and has been highlighted frequently. In four night shifts I have taken no breaks at all—should according to the British Medical Association be getting 90 minutes of breaks per 12-hour shift so have done 6 hours extra work in four nights and it's just not sustainable or safe for the patients”
“On-call shifts tend to get busiest from 19:00 onwards…it may be worth changing the hours of the on-call shifts in future to take the pressure off the surgical night team”
“Unable to safely take breaks/rest on night shift…as too many patients to clerk. It would not have been safe to leave them waiting…to be seen”
“Inability to take…breaks on night shift. This is a longstanding problem in the department and directly related to staffing. Overnight, the provision of staffing is often inadequate to deal with the influx of referrals and the management of sick ward patients”
“Overnight on-call/cover shift…Worked through breaks as the sole doctor on the ward with sick patients. The CST was busy with on-call activity away from the surgical assessment unit”
“High demand of patients…patients who arrived at 18:00 were not seen until 04:00. As a result I did not take any break. I was unsure how to resolve the matter during the night whilst not compromising patient safety due to delay in being seen”
“Ongoing unsustainable workload for surgical CST at night. The impression is that without covering T&O at night it would be manageable”
“Usual night time problems with too much work and the CST stretched across too many specialities”

The emergence of the COVID-19 pandemic in 2019 and its sequential “waves” correlated with significant variation in levels of Exception Reporting-they tended to drop particularly during the two main COVID-19 waves and respective periods of redeployment. This was acknowledged as an important confounder and impeded our ability to perform a valid analysis of the trends in ERs as a direct result of our intervention. The data demonstrated a slight downward trend in Exception Reporting from 2019 to 2022, correlated with improving conditions following this project. However ongoing issues with staffing particularly during day shifts continue to be a problem.

Between March 2019 and July 2021, prior to the intervention, there were 18 ISCs generated from within surgical specialities including T&O. Of these 63% were generated out-of-hours-11 (58%) were raised overnight and one (5%) at the weekend.

Between August 2021 and July 2022 there were nine ISCs generated within these specialities. Of these, 33% were generated out-of-hours-two (22%) were raised overnight and one (11%) at the weekend. The two ISCs raised overnight were within the General Surgery department, one because a locum doctor did not have access to IT systems and the second because there was no doctor available at all overnight, due to last-minute sickness. Overall, there was a downward trend in ISCs demonstrated before and after the change, particularly during OOH periods.

## Discussion

“Emergency cross-cover” is a term used to describe situations where OOH care is routinely provided by a doctor not normally working or training in the speciality, such as described in our Trust [5]. It occurs widely across the National Health Service (NHS) and can have implications for both patient safety and doctor wellbeing. Emergency cross-cover became more widespread following the introduction of the European Working Time Directive (EWTD), which limited weekly working hours, shift duration and number of consecutive shifts [6]. The EWTD led to increased shift-based working patterns, thus arguably resulting in decreased continuity of care for patients [5,7]. The concurrent implementation of Modernising Medical Careers, a restructuring of postgraduate medical training in the UK in 2005, further compounded the issue, as it significantly reduced the numbers of non-training Trust Grade doctors, including in our Trust [8].

Although cross-cover of specialities OOH is common, it can impact patient care and doctor training [5]. It is particularly prevalent in smaller specialities such as ENT, where its implications are widely discussed in the literature. Patients admitted with ENT emergencies OOH in the UK are likely to be initially assessed and managed by a doctor with little or no previous ENT experience, and doctors covering ENT OOH generally report low levels of confidence in managing ENT emergencies [5,9-11]. This is in contrast to the British Medical Association (BMA) guidance, which emphasises that doctors should not be required to cross-cover other specialities unless they have adequate competencies and training in those specialities [12]. Emergency cross-cover in General Surgery and T&O OOH is less frequently described in the literature and a fairly uncommon arrangement.

A summary of the potential problems associated with emergency cross-cover was published by ASIT in 2013 [5]. They described the legal and ethical implications of cross-cover, emphasising that it must always enable “safe, appropriate and timely patient care when needed.” Their investigation of a cohort of General Surgery trainees who were required to cross-cover Urology OOH repeatedly highlighted concerns related to insufficient supervision and inadequate training, which again is a breach of BMA recommendations [5,12]. Similar sentiments were echoed by our own trainee doctors when surveyed.

Our survey also highlighted the general theme of an overwhelming workload, which limits learning and training opportunities. All respondents who covered surgery OOH prior to August 2021 reported having felt unsafe, and in an era of ever-increasing reports of doctor burnout and poor mental health among healthcare staff, this stark statistic reflected an urgent need for intervention [13].

Staff shortages in healthcare are not a new problem; they have been described since the very conception of the NHS [14]. Newer phenomena continue to exacerbate the problem, including peak numbers of doctors taking time out of training or taking jobs overseas, increasing competition ratios in speciality training numbers and, more recently, effects of the COVID-19 pandemic [15]. The solution proposed by this Trust took into account some of the theories that have been postulated by creating job roles for non-training Trust Grade doctors with attractive incentives, including 40% non-clinical time commitment with opportunities for teaching, further education and dedicated theatre time [16]. It was recognised that with the disruption of COVID-19, fewer overseas posts were likely to be taken up by FY2 doctors, thereby creating a larger than normal pool of UK-trained doctors available for these posts, enabling the recruitment of excellent candidates.

The qualitative findings from this study demonstrate an overwhelmingly positive impact. Trainee doctors in surgical specialities are less overwhelmed by OOH and feel training opportunities and break times are more available to them. The quantitative data such as from Exception Reporting are less clear-cut, with ongoing concerns being raised at all levels. This would suggest that despite the significant increase in staffing levels OOH since the intervention in August 2021, there are further improvements that could be made in terms of staffing and supervision within surgical specialities within the Trust.

In light of the COVID-19 pandemic, the ever-increasing complexity of hospital patients and doctor burnout, there is clearly more work to be done; but this project marks the first steps in moving towards a safer OOH service for our patients and our doctors.

## Conclusions

In an ever-evolving, complex healthcare setting, historic short-term fixes such as the reallocation of Foundation Doctors to provide service provision within other departments can have detrimental long-term effects. The findings of our preliminary data collection from ERs, ISCs and anonymised surveys revealed multiple issues related to patient safety, junior doctor workload and training opportunities. By using this data, and enlisting the help of multiple valued senior stakeholders, an acceptable Business Plan was approved by the Trust with a view to reversing these issues. This improvement project has shown that the use of data, both quantitative and qualitative, to demonstrate longstanding problems can help advocate for funding and systemic change, improving the welfare of patients and doctors alike.
